# DNA–Polyelectrolyte Composite Responsive Microparticles for Versatile Chemotherapeutics Cleaning

**DOI:** 10.34133/research.0083

**Published:** 2023-03-15

**Authors:** Chong Wang, Jiali Wang, Zhuohao Zhang, Qiao Wang, Luoran Shang

**Affiliations:** Shanghai Xuhui Central Hospital, Zhongshan-Xuhui Hospital, and the Shanghai Key Laboratory of Medical Epigenetics, the International Co-laboratory of Medical Epigenetics and Metabolism (Ministry of Science and Technology) Institutes of Biomedical Sciences, Fudan University, Shanghai, 200032, China.

## Abstract

Drug therapy is among the most widely used methods in disease treatment. However, there remains a trade-off problem between drug dosage and toxicity. Blood purification by adsorption of excessive drugs during clinical treatment could be a solution for enhancing therapeutic efficacy while maintaining normal body function. Here, inspired by the intrinsic action mechanism of chemotherapeutic agents in targeting DNA in the cell nucleus, we present DNA–polyelectrolyte composite responsive microparticles for chemotherapeutics cleaning. The presence of DNA in the microparticles enabled the adsorption of multiple common chemotherapy drugs. Moreover, the microparticles are endowed with a porous structure and a photothermal-responsive ability, both of which contribute to improved adsorption by enhancing the contact of the microparticles with the drug solution. On the basis of that, the microparticles are integrated into a herringbone-structured microfluidic chip. The fluid mixing capacity and the enhanced drug cleaning efficiency of the microfluidic platform are validated on-chip. These results indicate the value of the DNA–polyelectrolyte composite responsive microparticles for drug capture and blood purification. We believe the microparticle-integrated microfluidic platform could provide a solution for settling the dosage–toxicity trade-off problems in chemotherapy.

## Introduction

Drug therapy is among the most efficient treatment for many diseases [1,2]. In general, to achieve a sufficient therapeutic effect, the dosage of the drug is usually excessive, which often causes systemic toxicity. To solve this, various delivery systems were constructed with the use of nano/microdrug carriers for targeted or topical administration [3–6]. Despite the great progress in this respect, the complex fabrication process of the carriers and the unresolved toxicity hinder the clinical translation of these delivery systems. Alternatively, materials and devices that can remove excessive drugs in serum promise to maximize therapeutic efficacy by avoiding severe side effects on healthy cells and tissues [7]. To this end, several drug capture materials or filters have been developed that rely on drug-specific capture materials for chemotherapeutics removal [8–11]. However, the practical application of drug-cleaning platforms requires a systematic design of the devices in terms of drug-binding mechanisms, structural optimization of the capture material, and well-defined adsorption dynamics, which are currently lacking. Thus, novel drug-cleaning platforms are still in urgent need.

**Fig. 1.**
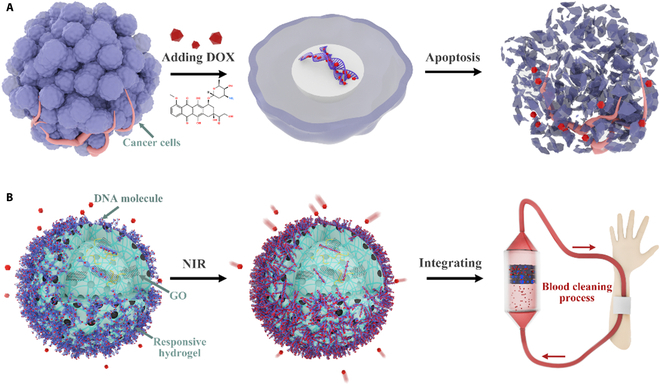
Doxorubicin (DOX) adsorption principle. (A) Schematic diagram of the action mechanism of a typical chemotherapy agent—DOX in living cells. (B) Schematic diagram of the DNA–polyelectrolyte composite responsive microparticles for chemotherapy agent adsorption and the integration of the microparticles into a device for blood cleaning. NIR, near infrared; GO, graphene oxide.

Here, inspired by the action mechanism of typical chemotherapy agents in living cells, we propose DNA-based responsive microparticles for chemotherapeutics cleaning, as shown in Fig. 1. DNA not only stores genetic information but also possesses excellent molecular recognition properties and unique structures, making it possible to construct functional materials such as DNA origami, DNA-functionalized nanoparticles, DNA–polymer conjugates, etc. [12–20]. Although DNA-based materials have been widely used in the biomedical field, a large portion of them focus on the assembly of a certain length of synthetic DNA sequences, while less is explored in utilizing natural DNA. Considering the intrinsic action mechanism of some chemotherapeutics in targeting DNA within the cell nucleus, the construction of genomic DNA-based materials is a facile and promising strategy for chemotherapeutics capture [9]. Responsive hydrogels are smart materials that could adapt to the surrounding environment and convert external stimuli into detectable signals [21–25]. In particular, responsive microparticles have been employed as microcarriers for the adsorption of water contaminants and disease biomarkers, while their value in the absorption of blood drugs has not yet been explored [7,26–28]. It is thus conceived that, by constructing DNA-based responsive microparticles, novel life-like chemotherapeutics capture materials could be developed.

In this paper, we construct DNA–polyelectrolyte composite microparticles (DPCMs) with photothermal-responsive properties and integrate these microparticles into a microfluidic mixer for versatile chemotherapeutics cleaning. Emulsion droplet templates containing methacrylate gelatin (GelMA), N-isopropyl acrylamide (NIPAM), graphene oxide (GO), and a polycation, poly(allylamine) (PAH), are first generated. The GelMA is mixed into the pregel solution to enhance the mechanical properties of the hydrogel microparticles [29]. By adopting gradient-cooling cryogelation and photopolymerization, microparticles with a porous structure are formed. Genomic DNA molecules are then assembled onto the microparticles via electrostatic complexation between DNA and PAH. The resultant microparticles could capture a series of chemotherapeutic agents including doxorubicin (DOX), epirubicin (EPI), and cisplatin (CIS) based on their specific binding between DNA. Moreover, the presence of Poly (N-isopropyl acrylamide) (PNIPAM) and GO enables the microparticles to undergo periodic shrinking and swelling under cycling irradiation of near infrared (NIR), which greatly promotes the process of mass transfer during drug adsorption. More interestingly, by embedding the microparticles into a herringbone-structured microfluidic device, improved adsorption capacity is achieved, owing to the enhanced contact between the microparticles and the drug solution. These results indicate that the DNA–polyelectrolyte composite responsive microparticles are smart chemotherapeutics capture materials and the present microfluidic-based platform holds promising potential in clinical drug cleaning and cancer therapy.

## Results

### Responsive hydrogel microparticles

**Fig. 2.**
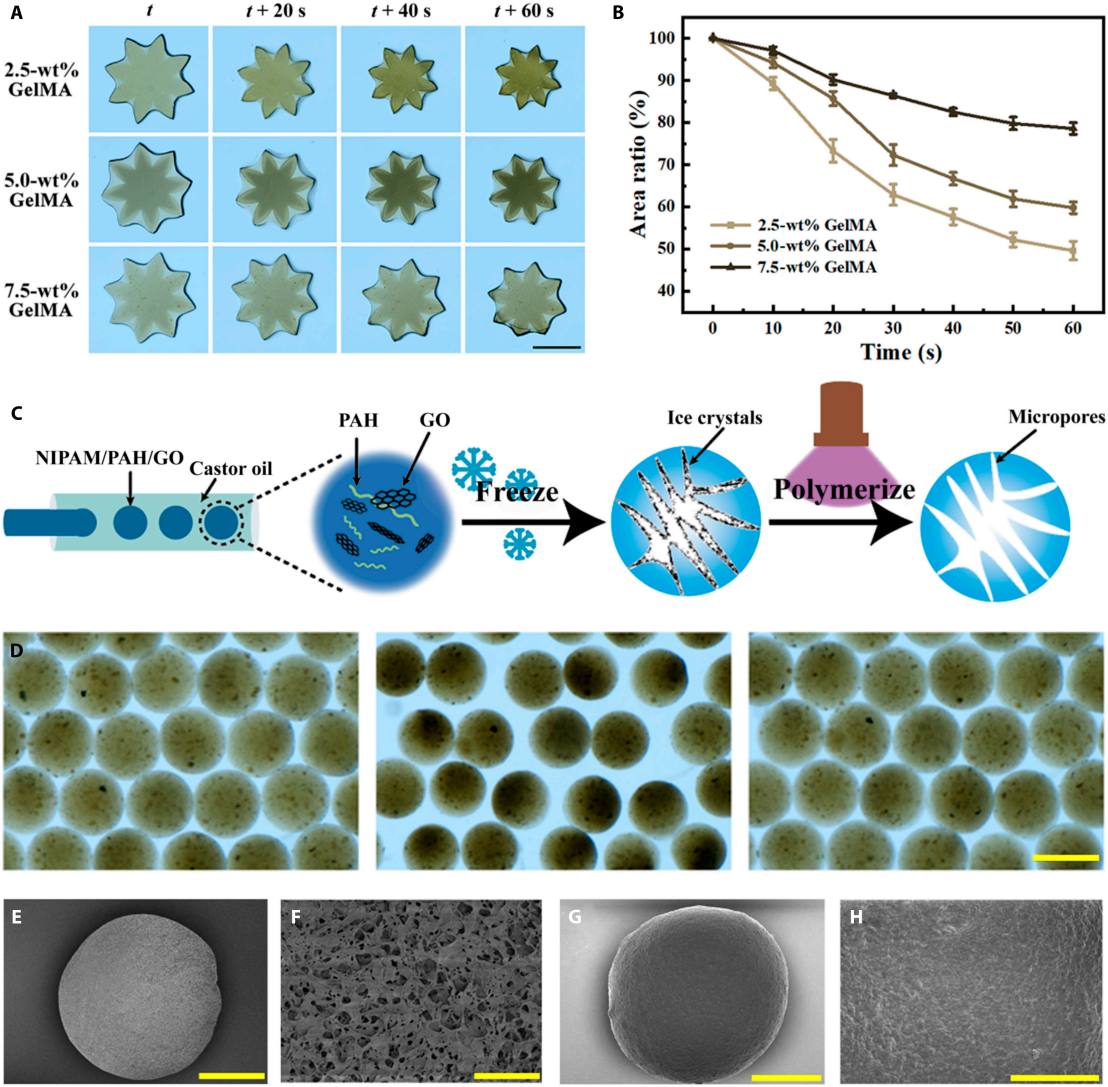
Characteristics of hydrogel microparticles. (A) Thermal-triggered shrinkage of the 8-angle star-shaped hydrogel film when heated under 40 °C at different times. Scale bar, 5 cm. (B) The change of the composite hydrogel film area with methacrylate gelatin (GelMA) concentrations of 2.5, 5, and 7.5 weight % (wt%) (*n* = 3). (C) Schematic diagram of the fabrication procedure of the polyelectrolyte composite responsive microparticles. (D) Optical microscope images showing the microparticles at the original state (left), under the irradiation of NIR (middle), and when the NIR light was switched off (right). Scale bar, 200 μm. (E) Scanning electron microscope (SEM) image of a single microparticle produced through gradient-cooling cryogelation. Scale bar, 100 μm. (F) SEM image showing the detail of the porous structure of the microparticle. Scale bar, 10 μm. (G) SEM image of a single PNIPAM/GelMA/GO composite microparticle generated by UV cross-linking readily after droplet generation. Scale bar, 100 μm. (H) SEM image showing the detail of the smooth surface of the microparticle. Scale bar, 40 μm. NIPAM, N-isopropyl acrylamide; PAH, poly(allylamine).

In a typical experiment, we first studied the stimuli-responsive behavior of the PNIPAM/GO/GelMA hydrogel. An 8-angle star-shaped hydrogel film was fabricated through ultraviolet (UV) polymerization with the aid of a photo mask. It was found that, with the temperature increased from room temperature to 40 °C, the hydrogel film shrank significantly within 1 min (Fig. 2A). This change indicated that the composite hydrogel has excellent temperature responsiveness, which was an important property for the derived responsive microparticles. Moreover, because GelMA is not temperature responsive, too much content of GelMA can suppress the deformation of the composite hydrogel upon heating. Thus, we compared the response of a series of hydrogel films derived from hydrogel precursor solutions containing different concentrations of GelMA monomer. We found that, for a same time of heating, the degree of shrinkage of the film was inversely related to the GelMA content in prepolymer solution (Fig. 2B). Therefore, we chose 2.5 weight % (wt%) as the optimized concentration in the following experiments.

On the basis of the optimized hydrogel formulation, we generated PNIPAM/GO/GelMA composite microparticles with uniform size in a glass capillary microfluidic chip with a co-flow channel geometry (Fig. 2C) [27,30,31]. The inner phase fluid was the hydrogel precursor solution, and the outer phase was castor oil. As we flowed the 2 fluids into the microfluidic device, monodispersed droplets of the prepolymer solution were produced as a result of the shear force between the 2 fluids and the interfacial tension (Fig. S1A). The coefficient of variation of the droplets was less than 5% (Fig. S1B). By shining UV light, the droplets were converted into solid microparticles. Note that the diameter of the cross-linked microparticles could be easily adjusted by the flow rates of the 2 phases. When the outer flow rate was constant, by increasing the inner phase flow rate, the diameter of the resultant microparticles increased accordingly (Fig. S1C). Conversely, under the constant inner flow rate, the diameter decreased with the increasing outer phase flow rate (Fig. S1D).

Considering the combination of the photothermal GO and thermal-responsive feature of PNIPAM, we explored the NIR-triggered contraction of the microparticles. As shown in Fig. 2D, under the NIR irradiation with constant power (1 W cm^−2^), the microparticles shrank by almost 50% and became dark because the water was squeezed out. Once the NIR was turned off, the microparticles returned to their original state. Such reversible behavior of volume change was conducive to the mass transfer between the microparticles and the surroundings. Moreover, the NIR-responsive efficiency of the microparticles was evaluated as a function of the GO concentration in the pregel solution. As the concentration of GO increased from 0.4 mg ml^−1^, the volume shrinkage rate increased in a positive correlation, but this trend was no longer obvious when the GO concentration exceeded 2.0 mg ml^−1^ (Fig. S2A). Thus, an optimized GO concentration of 2.0 mg ml^−1^ was chosen. Moreover, under these optimized conditions, we tested the reversible volume change behavior of the microparticles under cyclic NIR irradiation (Fig. S2B), which indicated the good durability of the microparticles. These properties ensured that the microparticles were used as a robust adsorption carrier.

To increase the surface area of the microparticles, a gradient-cooling cryogelation strategy was adopted to the microparticles with a porous structure [32]. First, the pregel droplets were frozen at −20 °C for ice crystal growth; this step promoted the formation of the porous network. Then, the droplets were frozen in liquid nitrogen (−196 °C) to prevent ice crystals from expanding further. Finally, the pregel droplets were cross-linked under the UV light, forming microparticles with interconnected pores. We characterized the microstructure of the microparticles using a scanning electron microscope. As shown in Fig. 2E and F, generated by gradient cooling procedures, the microparticles showed porous morphologies at the surface. By contrast, microparticles generated by direct UV cross-linking possessed a smooth surface (Fig. 2G and H).

### Electrostatic interaction-induced assembly of DPCMs

**Fig. 3.**
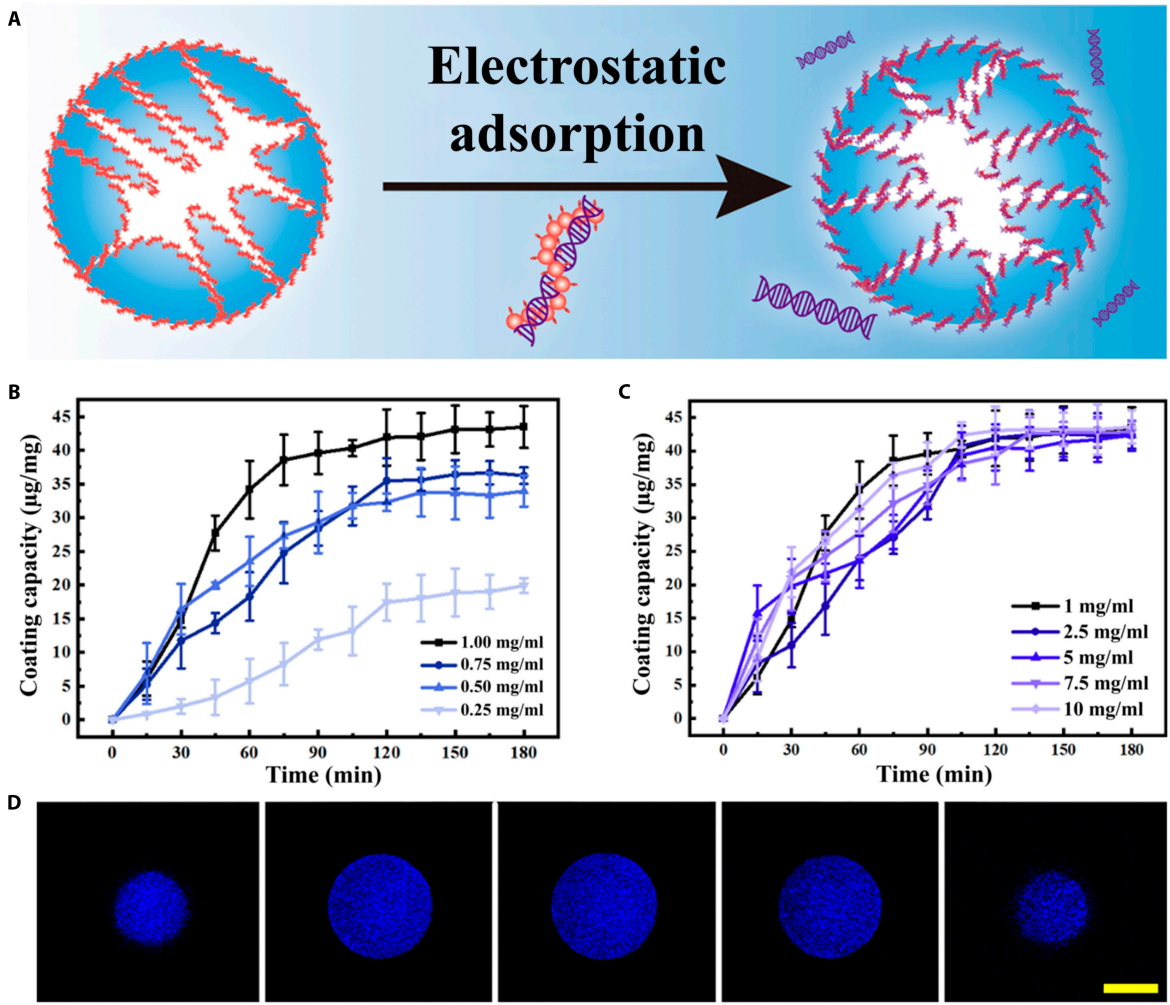
DNA coating capacity of composite microparticles. (A) Schematic diagram shows the coating principle of DNA onto the surface of microparticles. (B) The coating capacity of DNA as a function of PAH concentration in the pregel mixture and immersion time (*n* = 3). (C) The coating capacity of DNA as a function of DNA concentration and immersion time (*n* = 3). (D) A sequence of *z*-stack confocal laser scanning microscope images of the DNA–polyelectrolyte composite microparticles (DPCMs). The section depth is 20 μm. Scale bar, 100 μm.

To selectively capture chemotherapeutics, genomic DNA was incorporated into the microparticles through electrostatic interaction, as shown in Fig. 3A. This was achieved by adding PAH, a type of polycation, into the prepolymer solution to render the microparticles’ surface positively charged. The PAH-loaded microparticles were immersed in an excessive DNA solution to obtain DPCMs, and the DNA coating capacity was evaluated as the function of the concentration of the PAH contained in the prepolymer solution and the immersion time. As shown in Fig. 3B, after 180 min, the amount of DNA coated reaches the saturation point; as the PAH concentration in the prepolymer solution increases, the coating capacity increases accordingly. Therefore, we chose 1 mg/ml of PAH as the optimized concentration in subsequent experiments. Then, the relationship between the DNA concentration in the solution and the DNA coating capacity of the DPCMs was studied by immersing equal amounts of dried DPCMs into DNA solutions of different concentrations for 180 min. The result indicated that, when the DPCMs were immersed in DNA solution for 180 min, the coating capacity of DNA could reach a maximum of 43.6 ± 2.5 μg/mg (Fig. 3C). The successful coating of DNA was identified by energy-dispersive x-ray spectroscopy (Figs. S3 and S4). Moreover, after 4′,6-diamidino-2-phenylindole staining, the *z*-stack fluorescent images of the DPCMs indicated the uniform coating of DNA (Fig. 3D). On the contrary, microparticles with the absence of PAH showed no fluorescence (Fig. S5).

### The dynamic adsorption behavior of DPCMs

**Fig. 4.**
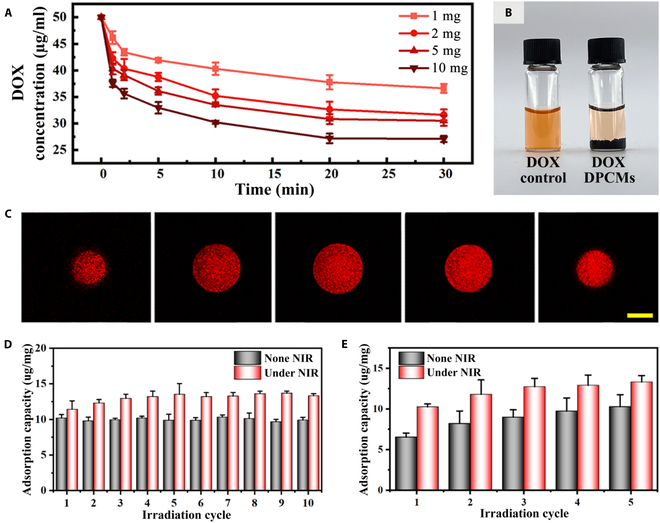
DOX adsorption capacity of DPCMs. (A) DOX capture with different amounts of DPCMs (*n* = 3). (B) The picture shows the color of the DOX solution (0.05 mg/ml) at the initial state (left) and after 10 mg of DPCMs was added for 30 min (right). (C) *Z*-stack confocal laser scanning microscope images of the DPCMs. The section depth was 20 μm. Scale bar, 100 μm. (D) The DOX adsorption capacity of DPCMs with or without periodic NIR irradiation. One milligram of DPCMs was pre-immersed in 1 ml of DOX solution (50 μg/ml) for 30 min (*n* = 3). (E) The adsorption capacity of DPCMs to DOX with or without periodic NIR irradiation (*n* = 3). The DPCMs were not pre-immersed in the DOX solution.

We next systematically tested the capture ability of DPCMs toward DOX. By immersing different amounts (1, 2, 5, and 10 mg) of DPCMs into 1 ml of DOX solution (50 μg/ml) for 30 min with constant stirring, their adsorption capacity was measured. As shown in Fig. 4A and B, for 10 mg of DPCMs added in the solution, the adsorption rate reached 46 ± 0.59% within 30 min. The absorption of DOX onto the DPCMs was further verified by fluorescence microscopy (Fig. S6), and confocal laser scanning microscope images demonstrated uniform adsorption (Fig. 4C). In contrast, the surface of the microparticles without coating DNA molecules showed negligible red fluorescence after immersing in the same DOX solution for 30 min (Fig. S7), which might be due to the physical adsorption; it also proved that the existence of DNA contributed to specific adsorption of DOX. Moreover, to study the effect of NIR stimulation on the drug adsorption process, we first immersed the DPCMs into a 50 μg/ml of DOX solution for 30 min to reach the static saturated state and then divided them into 2 groups: one that received NIR irradiation lasting for 1 min in every 6 min as the experiment group and the other without NIR irradiation as the control group. It was obvious that periodic NIR irradiation can further increase the adsorption capacity by about 34.4 ± 9.6%. This could be attributed to the periodic shrinking and swelling of the DPCMs. Compared with static adsorption, the dynamic behavior of the DPCMs under periodic NIR irradiation can make the interior of the microparticle fully contact with the solution, thus increasing the chance for DNA molecules inside the microparticles to adsorb DOX in the solution (Fig. 4D) [33]. In addition, another comparison experiment was performed to investigate the influence of NIR irradiation on the DOX adsorption capacity of DPCMs without pre-immersion. It shows that, compared with the non-NIR stimulation group, the adsorption time required for the DPCMs under NIR stimulation to reach the relatively maximum adsorption value was shortened by about 12 min. It was found that the periodic NIR stimulation not only accelerated the drug adsorption process of the DPCMs but also increased their maximum adsorption capacity Fig. 4E. These results indicated that the photoresponsive DPCMs are excellent adsorbents for chemotherapeutics capture.

### DPCM-integrated microfluidic platform

**Fig. 5.**
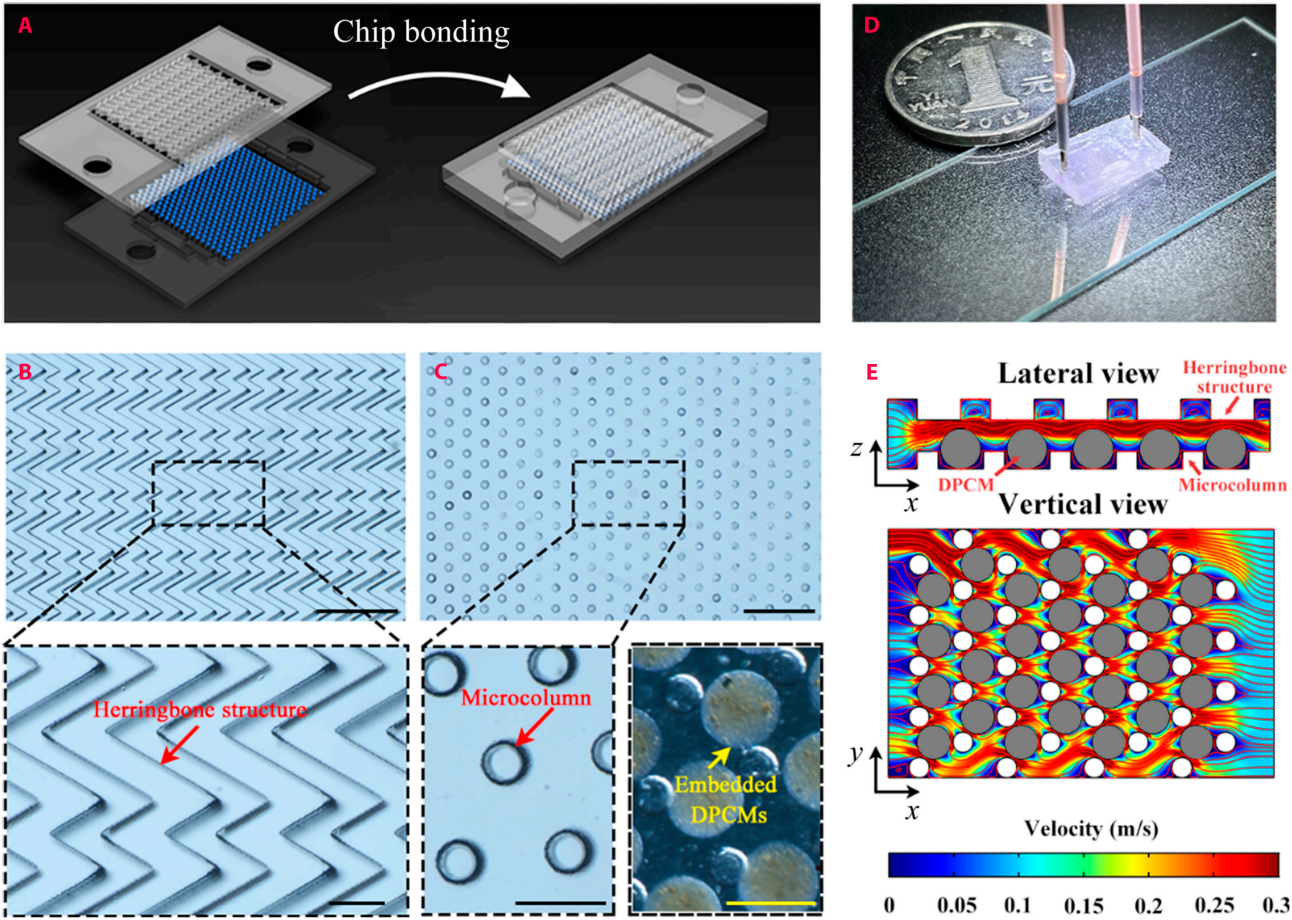
Characteristics of herringbone microfluidic chip. (A) Schematic diagram of the bonding process of the herringbone microfluidic device. (B) Optical microscopic images of the top layer containing herringbone-structured microfluidic channels with periodic microgrooves. Scale bars, 1 mm (top) and 250 μm (bottom). (C) Optical microscopic images of the bottom layer containing microcolumn arrays. The DPCMs were embedded in between. Scale bars, 1 mm (top) and 200 μm (bottom). (D) Photograph of a DPCM-integrated herringbone-structured microfluidic chip. (E) The vertical and lateral views of the numerical simulation of the flow in the DPCM-integrated device.

To further construct a chemotherapeutics cleaning platform, the as-prepared DPCMs were embedded in a polydimethylsiloxane (PDMS) microfluidic mixer. The microfluidic mixer was constructed by bonding a bottom layer containing microcolumns and a top layer containing herringbone-structured microchannels, as shown in Fig. 5A. The herringbone layer consists of patterned microgrooves oriented at an angle of 60°, as shown in Fig. 5B. The angle, length, height, and thickness of the herringbone structure were specially designed to match that of the microcolumns and microparticles. DPCMs were embedded within the microcolumn chip and separated individually, as shown in Fig. 5C. The periodic grooves were designed to enhance the turbulent flow of a fluid flowing through the device and, thus, increase the contact between the DPCMs and the fluid [34–36]. The constructed DPCM-integrated herringbone-structured microfluidic chip is shown in Fig. 5D. To confirm the enhancing mixing effect, we operate a numerical simulation to investigate fluid streamlines in the microfluidic device. As shown in the velocity streamline diagrams in Fig. 5E, the fluid streamlines seem distorted and rotated. These results indicated that the microfluidic device could generate chaotic advection of a fluid flowing through it.

**Fig. 6.**
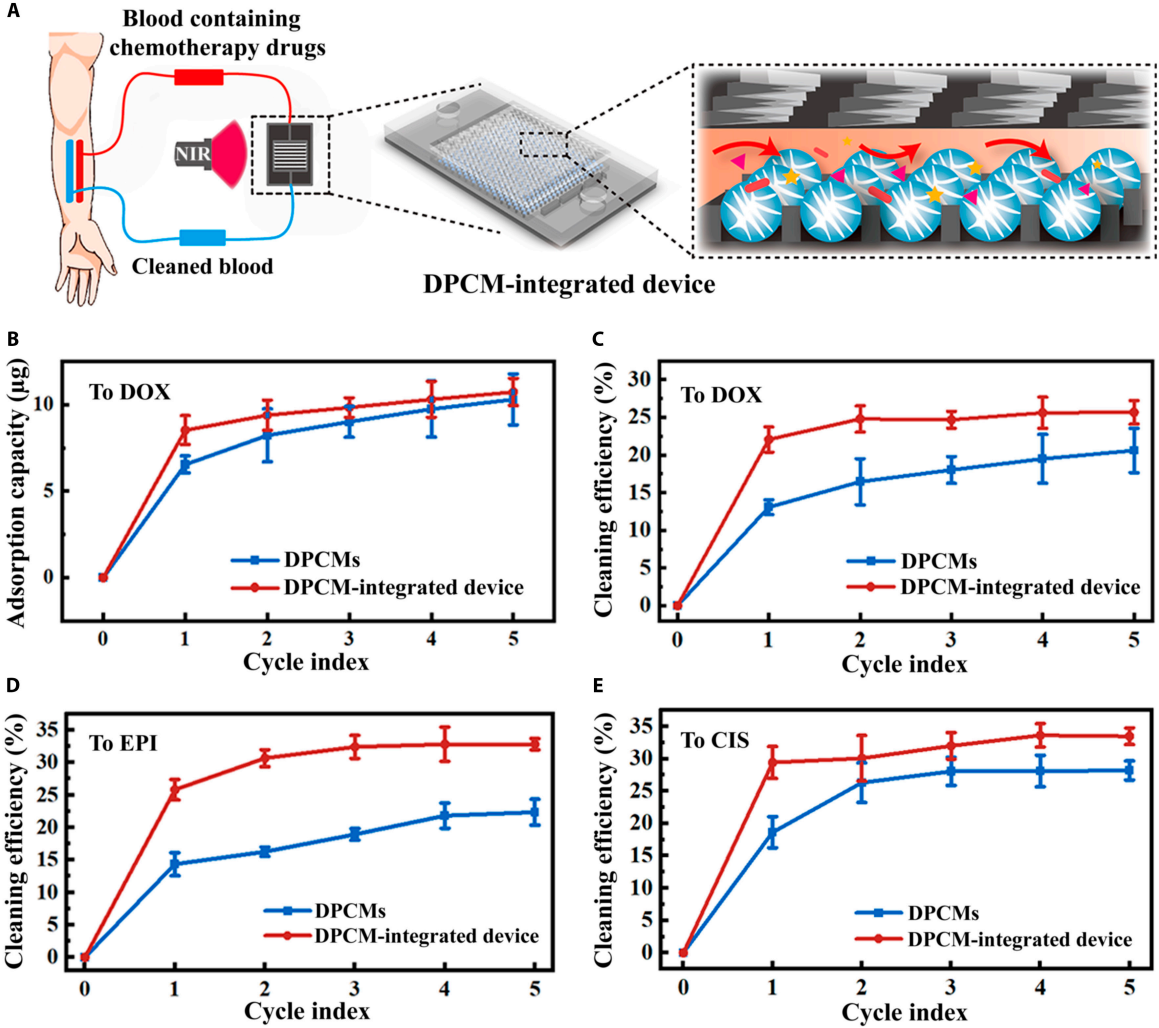
Chemotherapeutics cleaning capacity of DPCM-integrated microfluidic device. (A) Schematic diagram showing the particle-integrated microfluidic platform for dynamic and versatile cleaning of the chemotherapeutic agents. (B) The DOX adsorption capacity of the DPCM-integrated microfluidic device and bare DPCMs (*n* = 3). (C to E) Plots of the dynamic adsorption efficiency of (C) DOX, (D) EPI, and (E) CIS using the DPCM-integrated microfluidic device and combining with NIR stimulation (*n* = 3). The cycle index numbers represent both NIR irradiation cycles and solution injection cycles.

On the basis of the above results, we proposed a DPCM-integrated device to achieve the efficient adsorption of excessive chemotherapy agents during the chemotherapy (Fig. 6A). Because the fluid advection could enhance mixing between the embedded DPCMs and the surrounding liquid, we further performed a quantitative study to investigate such effect on the adsorption capacity of the DPCMs toward DOX. As an experiment group, we first integrated DPCMs within the microfluidic device, and 1 ml of DOX solution (50 μg/ml) was injected into the device at a flow rate of 167 μl/min. Multiple adsorption cycles were conducted, and the total DOX adsorption was measured using a microplate reader as a function of the number of cycles. As a control, the same number of DPCMs were immersed in a 1-ml DOX solution (50 μg/ml) for 30 min to measure the adsorption capacity; the sample of the solution was taken every 6 min, corresponding to the time duration of one cycle on-chip. Compared with the control group, the adsorption of DOX in the DPCM-integrated microfluidic device was significantly faster (Fig. 6B). The results confirmed that the herringbone-structured microfluidic device facilitated the adsorption of small-molecule drugs by enhancing the contact between the drug solution and the DPCMs.

Because both periodic NIR stimulation and advection-induced mixing could promote the drug adsorption process of the DPCMs, we combined these 2 elements and proposed a dynamic adsorption strategy, which could be further developed as a cleaning equipment for the removal of excessive chemotherapeutic drugs. Specifically, we injected 1 ml of DOX solution (50 μg/ml) into the integrated device; in the meantime, 1 W cm^−2^ of NIR was periodically irradiated to the center of the microfluidic chip, and a centrifugal tube was placed at the exit of the chip to collect the solution after cleaning. The flow rate of DOX solution was set as 167 μl/min, and the NIR irradiation was performed 1 min in every 6 min (with 5-min interval), corresponding to the duration of one flow cycle. We tested the dynamic adsorption efficacy toward DOX and another 2 DNA-targeting chemotherapeutics, EPI and CIS, respectively. As shown in Fig. 6C to E, the efficiency of dynamic adsorption toward DOX, EPI, and CIS in the platform was significantly higher than that of static adsorption. These results indicated that the present platform provided an efficient and dynamic adsorption method for versatile chemotherapeutics cleaning.

## Discussion

In conclusion, we reported novel microparticles as adsorption agents for the cleaning of multiple chemotherapeutic drugs. Inspired by the action mechanism of chemotherapeutic drugs targeting DNA in cell nuclei, we prepared genomic DNA-functionalized hydrogel microparticles via electrostatic interaction with polyelectrolytes. The resultant microparticles were thus enabled to adsorb common DNA-targeted drugs, including DOX, CIS, and EPI. Besides, PNIPAM and GO were incorporated into the microparticles, by which the microparticles exhibited photothermal-responsive volume change upon NIR stimulation. Moreover, the microparticles were endowed with a porous structure through a gradient-cooling cryogelation strategy. These combined effects facilitated more contact of the microparticles with the drug solution and resulted in higher adsorption. Moreover, an integrated drug-cleaning platform was established by embedding the microparticles within a herringbone-structured microfluidic chip. The chaotic fluid advection and NIR-stimulated dynamic response of the microparticles largely enhanced fluid mixing and mass transfer, thus achieving a high adsorption efficiency to multiple chemotherapy drugs. These features make the microparticles excellent for removing excess chemotherapeutic drugs and for reducing their damaging side effects. The application of the DPCMs is not limited to chemotherapy drug adsorption. For example, by coating DNA probes, high-sensitivity detection of nucleic acids can be realized. In addition, the polyelectrolyte composite responsive microparticles may be used for DNA vaccine delivery and gene therapy. In like manner, carriers of DNA molecules are not limited to light- and heat-responsive hydrogel microparticles. Other types of functional hydrogels can also be used as carriers for chemotherapy drug clearance. We also envisage that the present microfluidic platform can be further extended into other types of drug capture and blood purification applications.

## Materials and Methods

### Materials

GelMA was obtained from Cure Gel Co. Ltd. NIPAM, PAH, N,N′-methylenebis(acrylamide), and photoinitiator 2-hydroxy-2-methylpropiophenone were bought from Sigma-Aldrich. Phosphate-buffered saline (PBS) was purchased from Wuhan Servicebio Technology Co. Ltd. DOX, EPI, and CIS powders were purchased from Aladdin. PDMS was obtained from Dow Corning. Ethanol was purchased from Sinopharm Chemical Reagent Co. Ltd. DNAs from salmon sperm were purchased from RHAWN.

### Generation of porous microparticles

A co-flow microfluidic device was assembled to fabricate composite microparticles through droplet templates [27,31]. An aqueous solution containing 10 wt% of NIPAM, 0.34 wt% of N,N′-methylenebis(acrylamide), 0.2 wt% of GO, 1 volume % of 2-hydroxy-2-methylpropiophenone, and 0.1 wt% of PAH was the inner phase, and castor oil was the outer phase. The 2-phase fluids were separately injected into the inner and outer tubes of the device with a typical flow rate of 0.1 and 1.5 ml h^−1^, respectively. The inner phase fluid was pinched off into monodispersed droplets under the action of shear force and surface tension force, and the droplets were finally collected in a container. After freezing at −20 °C for 30 min, the obtained droplets were moved into liquid nitrogen, and subsequent UV irradiation was performed to polymerize the droplets. Once the cross-linking was accomplished, the microparticles were washed with alcohol to clean the surrounding castor oil.

### Generation of DPCMs

The as-prepared microparticles were immersed in an aqueous solution of DNA (1 mg/ml) for 180 min. After that, the content of DNA remaining in the solution was measured by NanoDrop. The DNA coated onto the microparticles was calculated by the following equation: *a* = (*c*_0_ − *c*_1_) × *V*/*w*, where *a* refers to the mass of DNA coated on the microparticles (per milligram), *c*_0_ indicates the initial concentration of DNA, *c*_1_ refers to the final concentration of DNA, *V* indicates the total volume of DNA solution, and *w* indicates the mass of added microparticles. Excess DNA was washed away with PBS to obtain the DPCMs.

### Measurement of the adsorption capability of the DPCMs

#### DOX

One milligram of DOX was first dissolved in 1 ml of H_2_O, and 19 ml of PBS was added to dilute the concentration of DOX into 0.05 mg/ml. Then, different amounts of DPCMs (1, 2, 5, and 10 mg) were added to the solution to start the adsorption. Moreover, mechanical stirring was constantly applied during the adsorption process. At each time point, a 30-μl DOX solution was taken and diluted to 300 μl and subsequently placed in a 96-well microplate. Then, the concentration of DOX was measured using a microplate reader (excitation wavelength is 478 nm; emission wavelength is 580 nm). The adsorption capacity of DPCMs was calculated by the following equation: *D* = (*C*_0_ − *C*_1_) × *V*/*w*, where *D* refers to the total amount of small-molecule drugs captured by DPCMs (per milligram), *C*_0_ indicates the initial concentration of drugs, *C*_1_ refers to the final concentration of drugs in the solution, *V* indicates the total volume of drug solution, and *w* represents the total mass of added DPCMs.

#### EPI

Nineteen milliliters of PBS was added to a centrifuge tube first. Then, an aqueous EPI solution with a concentration of 1 mg/ml was added to adjust the concentration to 0.05 mg/ml. After that, DPCMs were added to the solution with constant stirring. At every time point, a 30-μl EPI sample is taken and diluted to 300 μl and subsequently placed in a 96-well microplate. Then, the concentration of EPI was measured by using the fluorescence on a microplate reader (excitation wavelength is 474 nm; emission wavelength is 557 nm). The calculation procedure was consistent with the above.

#### CIS

Nineteen milliliters of PBS was added to a centrifuge tube first. Then, an aqueous CIS solution with a concentration of 1 mg/ml was added to adjust the concentration to 0.05 mg/ml. Then, DPCMs were added to the solution to start the adsorption process. At each time point, a 50-μl CIS solution was taken and diluted to 5 ml. Then, the concentration of platinum remaining in the solution was measured by inductively coupled plasma mass spectrometry to determine the CIS concentration. The calculation procedure was consistent with the above.

#### DOX adsorption under cyclical NIR irradiation

One milligram of DPCMs was immersed into a 1-ml DOX solution (0.05 mg/ml). Then, 1 W cm^−2^ of NIR irradiation was applied to stimulate the dynamic shrinkage of the DPCMs for 1 min. After that, the NIR irradiation was turned off for 5 min. This periodic process was repeated 5 times. At the end of every cycle, a 30-μl solution sample was collected, and the DOX concentration was measured by a microplate reader with the method mentioned above.

### Construction of the DPCM-integrated microfluidic device

A PDMS microfluidic chip was constructed by bonding a top layer and a bottom layer. The top layer contained herringbone-structured microchannels and the bottom layer contained an array of microcolumns; both the top layer and the bottom layer were obtained by replicating corresponding 3-dimensional (3D) printed templates. PDMS curing agent was mixed with the base at a ratio of 1:10. After mixing well, the mixture was poured into the 3D printed resin mold and solidified at 70 °C for 1 h. Then, the solid PDMS layers were peeled off from the molds and treated with oxygen plasma for 3 min. Then, the DPCMs were placed at the bottom microcolumn layer, each was confined in between 3 microcolumns. Subsequently, the top layer was covered on top of the bottom layer, and a clamp was utilized to fix them. After being fixed in a vacuum environment for 30 min, the bonded chip was obtained.

### Measurement of the dynamic adsorption capability of the DPCM-integrated device

#### DOX

One milliliter of DOX solution (0.05 mg/ml) was prepared and injected into the DPCM-integrated device at a rate of 167 μl/min. Meanwhile, for NIR-stimulated adsorption, 1 W cm^−2^ of NIR was irradiated onto the chip for 1 min, from a distance of 10 cm. As the irradiation was completed, the microparticles were rested for 5 min. As mentioned above, to maximize the cleaning efficiency, 5 cycles of the cleaning process were conducted. At the end of each cleaning cycle, 30 μl of solution was collected to calculate the cleaning efficiency.

#### EPI

One milliliter of EPI solution (0.05 mg/ml) was prepared and injected into the DPCM-integrated device at the rate of 167 μl/min. Meanwhile, for NIR-stimulated adsorption, 1 W cm^−2^ of NIR was irradiated onto the chip for 1 min, from a distance of 10 cm. As the irradiation was completed, the microparticles were rested for 5 min. As mentioned above, 5 cycles of the cleaning process were implemented to maximize the cleaning efficiency. At the end of each cleaning cycle, 30 μl of solution was collected to calculate the cleaning efficiency.

#### CIS

One milliliter of CIS solution (0.05 mg/ml) was prepared and injected into the DPCM-integrated microfluidic device at the rate of 167 μl/min. Meanwhile, for NIR-stimulated adsorption, 1 W cm^−2^ of NIR was irradiated onto the chip for 1 min, from a distance of 10 cm. As the irradiation was completed, the microparticles were rested for 5 min. As mentioned above, the cleaning process was implemented for 5 cycles to maximize the cleaning efficiency. At each time point, a drop of 50 μl of solution was taken, and the adsorption capacity was measured according to the standard protocol.

### Numerical simulation

The numerical simulation was based on the 3D incompressible Navier–Stokes equation to investigate the fluid flow states in the microfluidic chip. The density of the fluid was set as 1,000 kg/m^3^. The viscosity was set as 1.01 × 10^−3^ Pa s. The flow rate of the model fluid was set as 167 μl/min. The temperature was set as 293.15 K.

### Characterization

The microstructures and energy-dispersive x-ray spectroscopy mapping of the microparticles were acquired using a scanning electron microscope (Regulus 8100, Hitachi). Optical and fluorescence images of microparticles were recorded using a stereoscopic fluorescence microscope (NSZ818, Nexcope) equipped with a charge-coupled device camera (E3ISPM, Suzhou Jingtong Instrument). The concentration of DNA was measured by NanoDrop One (Thermo Fisher). The concentration of DOX and EPI were measured with the microplate reader (BioTek Synergy 2). The concentration of CIS in the solution was measured with inductively coupled plasma mass spectrometry (ICAP-QC, Thermo Fisher).

## Data Availability

All data needed to evaluate the conclusions in the paper are present in the manuscript and the Supplementary Materials.
