# Supplementary material for: DNA–Polyelectrolyte Composite Responsive Microparticles for Versatile Chemotherapeutics Cleaning
Source: Research (Wash D C). 2023 Mar 15;6:0083. doi: 10.34133/research.0083 (PMC10017331; doi:10.34133/research.0083)
Supplement: Supplementary 1 — Figs. S1 to S7 [file research.0083.f1.docx]

Supplementary Materials for

**• DNA Polyelectrolyte Composite Responsive Microparticles for Versatile Chemotherapeutics Cleaning**

Chong Wang *et al.*

*Corresponding author: Luoran Shang, luoranshang@fudan.edu.cn

**This PDF file includes:**

Figs. S1 to S7


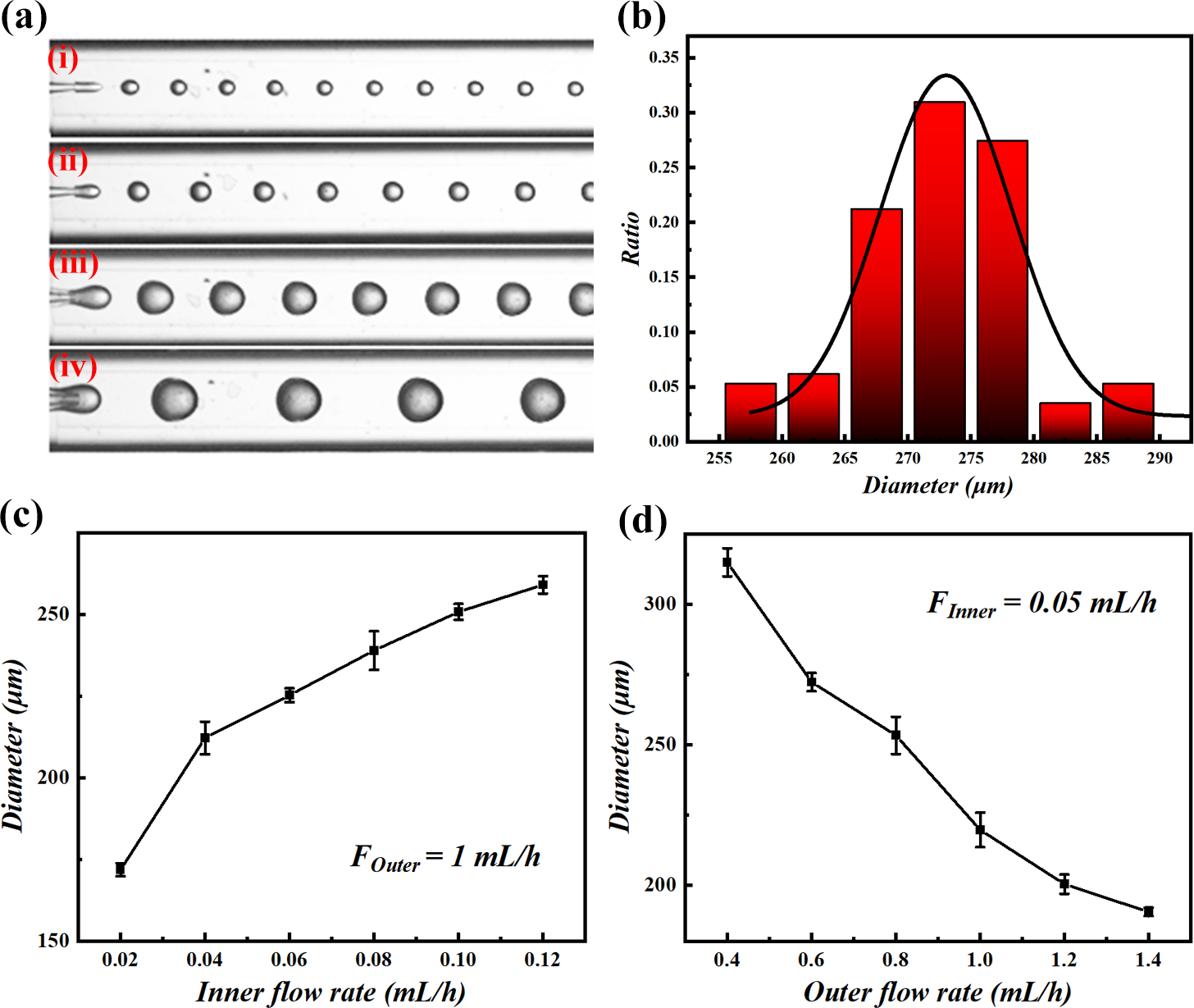


**Fig. S1.** **The characteristics of pregel droplets.** (**a**) The production process of droplet templates in the microfluidic chip. The flow rates of the droplet phase and the continuous phase are (i) 0.05 and 1.5 mL h^-1^, (ii) 0.05 and 1 mL h^-1^, (iii) 0.1 and 1 mL h^-1^, and (iv) 0.2 and 1 mL h^-1^, respectively. (**b**) The size distribution of the resultant droplets. (c, d) Plots of the diameter of the microparticles as a function of (**c**) the outer flow rate and (**d**) the inner flow rate (n = 3).


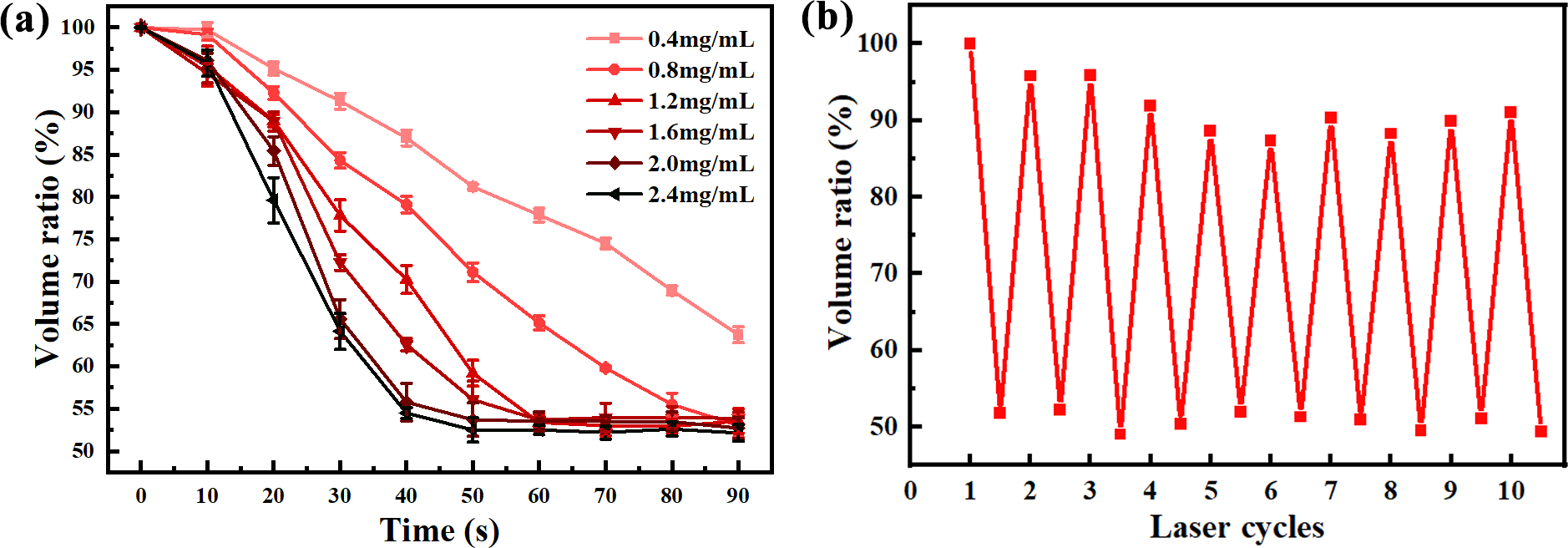


Fig. S2. Photo-thermal responsive ability of hydrogel microparticles. (a) The volume change of the microparticles with different concentrations of GO in the pregel mixture solution under the irradiation of NIR (1 W cm^−2^). (b) The volume change of the microparticles under cyclic NIR (1 W cm^−2^) irradiation.


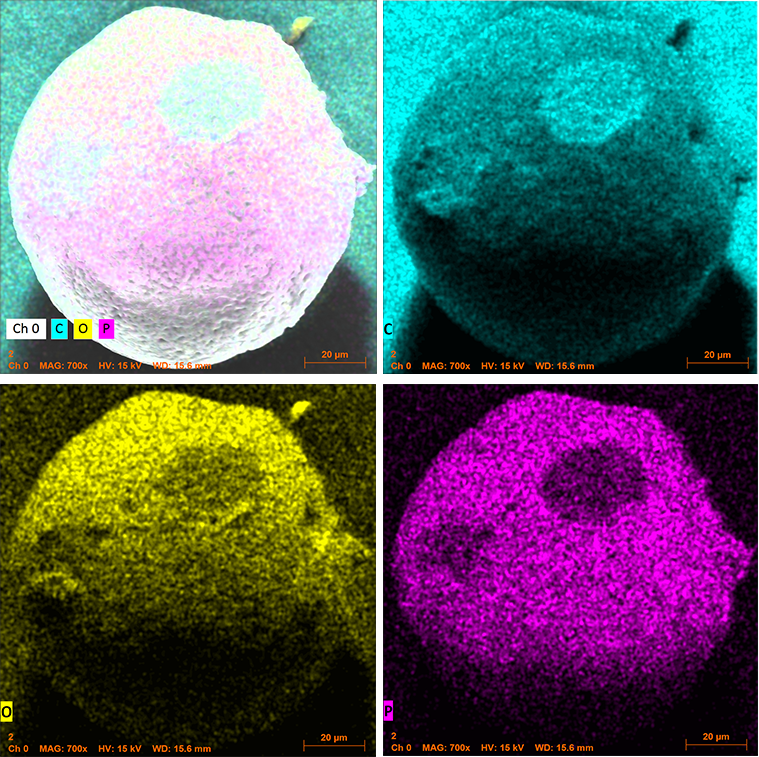


Fig. S3. EDS element maps of the DNA polyelectrolyte composite microparticles.


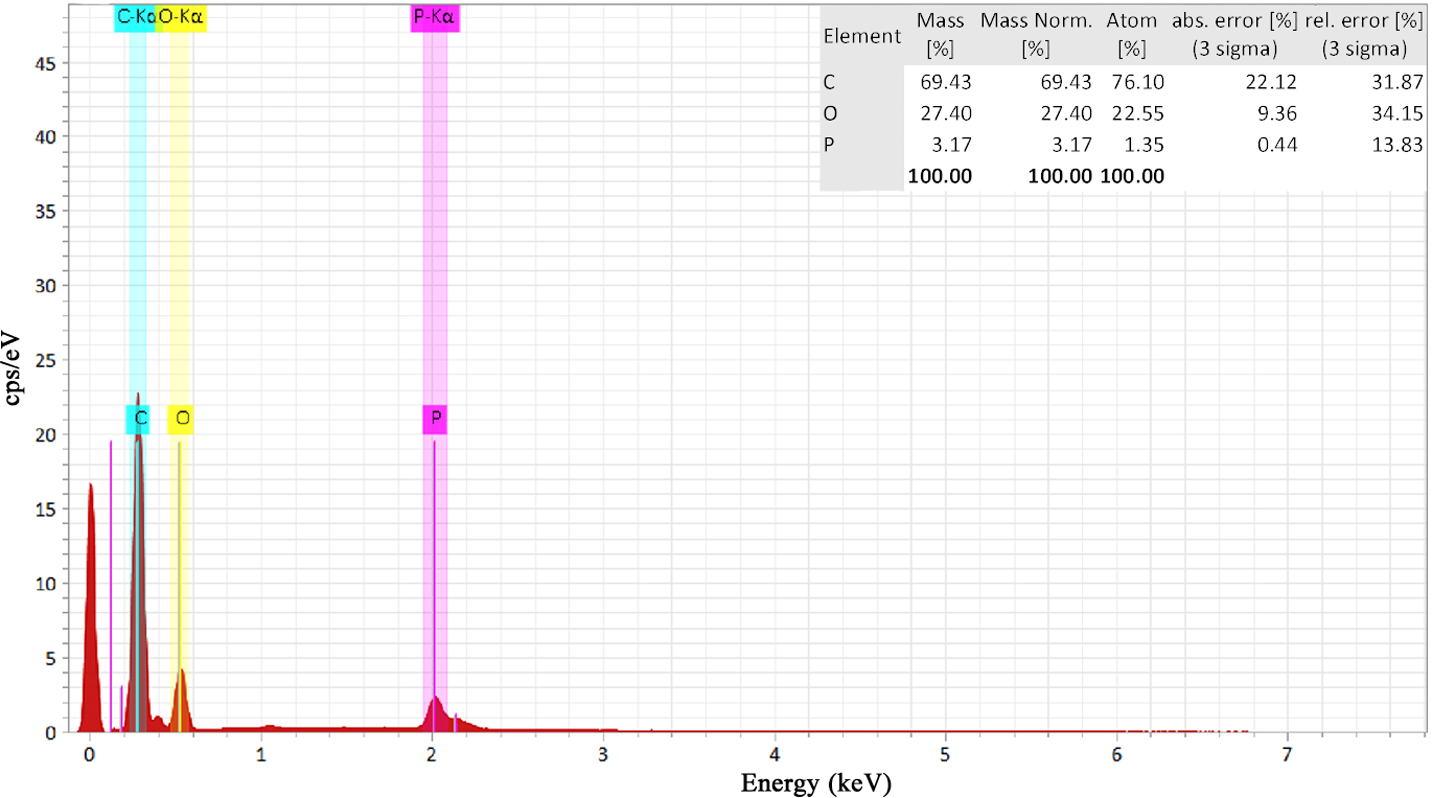


Fig. S4. EDS spectrum of the DNA polyelectrolyte composite microparticles.


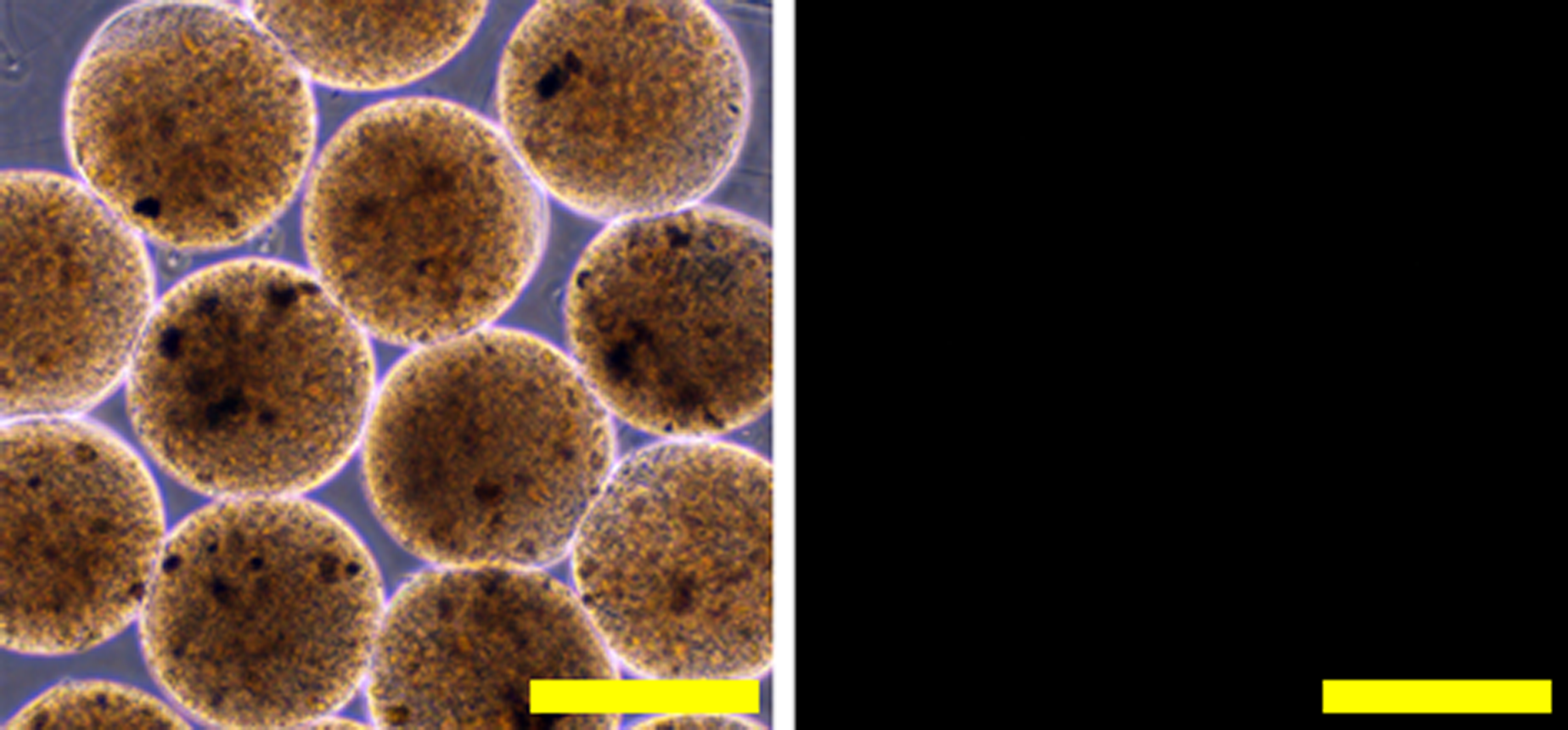


Fig. S5. Bright-field (left) and fluorescence (right) microscopic images of the microparticles without DNA coating. Scale bars are 200 µm.


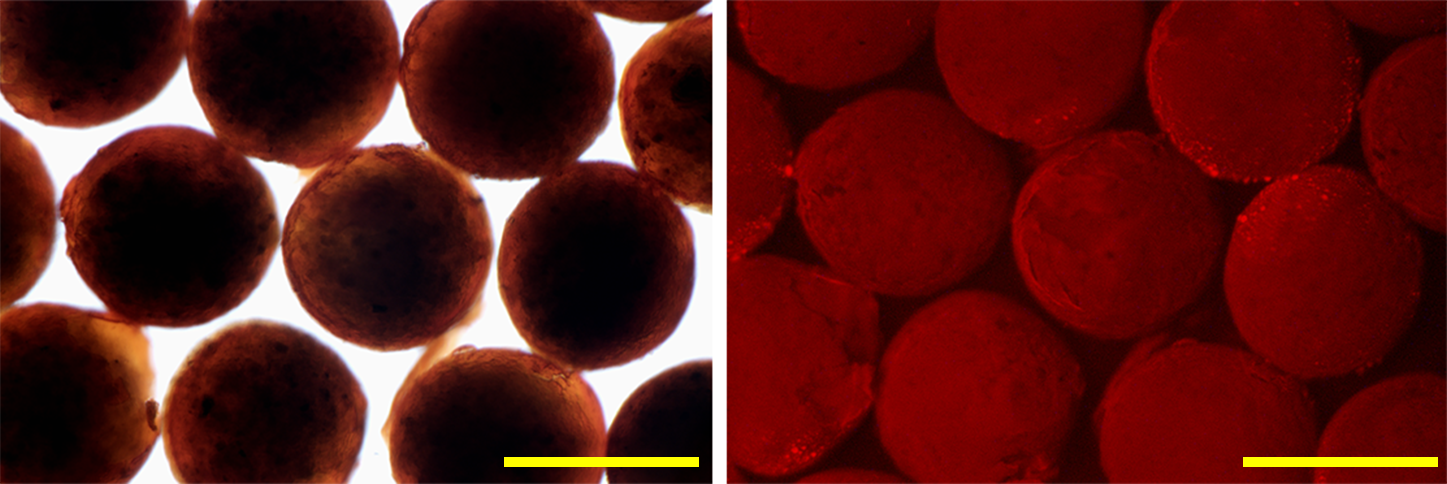


Fig. S6. Brightfield (left) and fluorescence (right) images of the DPCMs after adsorption of DOX. Scale bars are 200 μm.


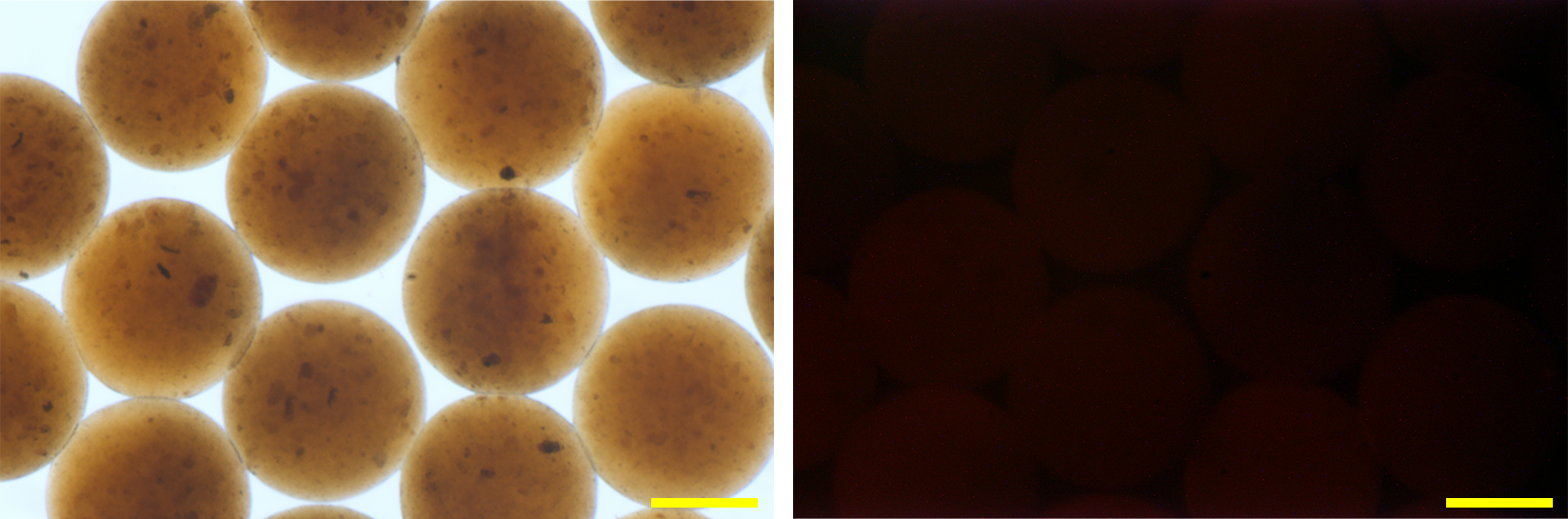


Fig. S7. Brightfield (left) and fluorescence (right) images of the microparticles without coating DNA after immersion in a DOX solution. Scale bars are 100 μm.
